# A Low-Cost Point-of-Care Testing System for Psychomotor Symptoms of Depression Affecting Standing Balance: A Preliminary Study in India

**DOI:** 10.1155/2013/640861

**Published:** 2013-09-24

**Authors:** Arindam Dutta, Robins Kumar, Suruchi Malhotra, Sanjay Chugh, Alakananda Banerjee, Anirban Dutta

**Affiliations:** ^1^The Gnan Systems LLP, Kolkata 700156, India; ^2^The Dharma Foundation of India, New Delhi 110003, India; ^3^The Neuro Rehab Services LLP, New Delhi 110048, India; ^4^The Charité-Universitätsmedizin Berlin, 10117 Berlin, Germany; ^5^Institut National de Recherche en Informatique et en Automatique (INRIA), 34090 Montpellier, France

## Abstract

The World Health Organization estimated that major depression is the fourth most significant cause of disability worldwide for people aged 65 and older, where depressed older adults reported decreased independence, poor health, poor quality of life, functional decline, disability, and increased chronic medical problems. Therefore, the objectives of this study were (1) to develop a low-cost point-of-care testing system for psychomotor symptoms of depression and (2) to evaluate the system in community dwelling elderly in India. The preliminary results from the cross-sectional study showed a significant negative linear correlation between balance and depression. Here, monitoring quantitative electroencephalography along with the center of pressure for cued response time during functional reach tasks may provide insights into the psychomotor symptoms of depression where average slope of the Theta-Alpha power ratio versus average slope of baseline-normalized response time may be a candidate biomarker, which remains to be evaluated in our future clinical studies. Once validated, the biomarker can be used for monitoring the outcome of a comprehensive therapy program in conjunction with pharmacological interventions. Furthermore, the frequency of falls can be monitored with a mobile phone-based application where the propensity of falls during the periods of psychomotor symptoms of depression can be investigated further.

## 1. Introduction

The World Health Organization (WHO) estimated that major depression is the fourth significant cause of disability for people aged 65 and above [1], where depression is a major contributor to the healthcare costs associated with the elderly population. Depression is a major health issue for elders, yet late-life depression often goes undiagnosed [2]. One in every four among India's elderly (age > 60 years) population is depressed, and around one in 10 experiences a fall that results in fracture [3]. In fact, the elderly population is predicted to increase to 12% of the total population by 2025 [3]. Depressed elderly report decreased independence, poor health, poor quality of life, functional decline, disability, and increased chronic medical problems [1]. Moreover, psychomotor symptoms of depression may contribute to falls among elderly and an associated fear of falling [4]. Here, course of depression, diurnal variation, medication status, gender, and age are associated with psychomotor agitation and retardation [5]. The psychomotor symptoms in depression have unique significance where they have high discriminative validity, may be the only symptoms of depression that distinguish depression subtypes, and are predictive of good response to medicines such as tricyclic antidepressants [5]. Therefore point-of-care testing (POCT) of the psychomotor symptoms of depression is urgently needed in order to screen community dwelling elderly at risk as well as to monitor the course of treatment [2], which takes 2 to 4 weeks to work fully, and a normal course lasts at least 6 months [5].

The costs of such screening of the psychomotor symptoms of depression is important in a low-resource setting, for example, in India, where a low-cost POCT of the psychomotor symptoms of depression was developed and evaluated in this study. In fact, Indian health ministry's working group on noncommunicable disease burden [3] noted that “India will soon become home to the second largest number of elderly in the world. The challenges are unique with this population in India.” In accordance, Kumar and colleagues at the Dharma Foundation of India (DFI) conducted a preliminary cross-sectional study on 95 elderly (age > 60 years) subjects from New Delhi and Gurgaon, India [6]. They investigated psychomotor aspects of depression where they found a negative correlation between the Berg Balance Scale (BBS) score [7] and the Geriatric Depression Scale (GDS) score [8]. Also, they found that as age increased, the BBS score decreased and the GDS score increased, leading to poorer balance and depression. Following this alarming insight, DFI conducted a subsampling of 24 community dwelling elderly from their database in India that covered a wide range of BBS and GDS scores, in order to evaluate a low-cost POCT [9] system for the psychomotor symptoms of depression. The first objective of this report is to present the low-cost POCT system for psychomotor symptoms of depression. The second objective is to present the results from the clinical study on 24 community dwelling elderly to evaluate the POCT system. It was hypothesized that the POCT system will be able to capture the psychomotor symptoms of depression in elderly.

## 2. Methods

### 2.1. Preliminary Cross-Sectional Study

Kumar and colleagues conducted a cross-sectional study [6] on 104 subjects, who were found through convenient sampling of community dwelling elderly in Delhi and Gurgaon, India. The inclusion criteria of the study were age of elderly 60 years and above, both males and females, and the subjects should have the ability to read and understand either Hindi or English. The exclusion criteria were severe problem with vestibular and visual system, any self-reported history of psychiatric illness excluding depression, cognitive deficits due to any reason, and use of assistive aids for walking, for example, wheel chair, crutch, walker, cane. The evaluation tools used in the study are described below.

- Hindi Mental State Examination (HMSE) [10, 11] was developed for the Hindi-speaking semiliterate and illiterate population of rural northern India. The total score possible was 31 where the cut-off score was 19.
- Berg Balance Scale (BBS) [7] consists of 14 common tasks. The 14 items are scored on a 5-point ordinal scale (0 = unable to perform, 4 = independent) based on ability to complete the timed tasks.
- Geriatric Depression Scale (GDS) [8] is a brief questionnaire in which participants are asked to respond to the 30 questions by answering yes or no in reference to how they felt on the day of administration. Scores of 0–9 are considered normal, 10–19 indicate mild depression, and 20–30 indicate severe depression.

Only 9 out of 104 subjects were excluded on the basis of the HMSE exclusion criteria, leading to a sample size of 95. The data was then analyzed by statistical software, as described further by Kumar and colleagues [6], where the descriptive statistics were calculated for the age, BBS, GDS, and frequency statistics of Gender. The Pearson correlation and regression analysis were applied to find out the relationship between age, BBS, and GDS. Independent sample *t*-test was applied to compare the scores of BBS and GDS between males and females.

### 2.2. Point-of-Care Testing (POCT) of Psychomotor Symptoms of Depression

Following the preliminary cross-sectional study by Kumar and colleagues [6], a convenient subsampling of the community dwelling elderly in India was conducted to select 24 elderly (>60 years) subjects, such that they covered a wide range of BBS and GDS scores. All subjects gave their informed consent for the experiments in compliance with the Helsinki Declaration.

**Figure 1:**
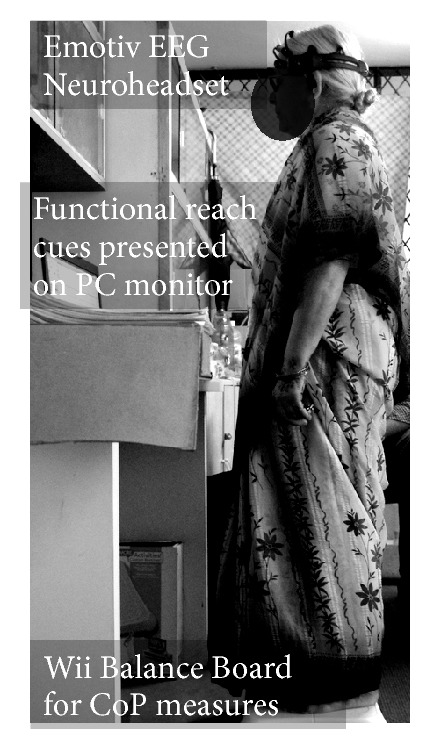
An illustration of the experimental setup.

The objective was to evaluate a low-cost POCT system to investigate the psychomotor symptoms of depression affecting standing balance. The experimental setup is shown in Figure 1. The centre of pressure (CoP) was monitored using a Wii Balance Board (Nintendo, USA) [13] and custom software developed using the WiiBrew toolkit [14]. The Wii Balance Board (Wii BB) relayed the 2D ground reaction force information to the PC via Bluetooth [14], while Emotiv EEG Neuroheadset (Emotiv, Australia) relayed EEG data to the PC via their proprietary wireless protocol [12]. The specifications of the EEG Neuroheadset [12] are detailed in the Table 1. 

#### 2.2.1. Real-Time Feedback of the Center of Pressure

**Figure 2:**
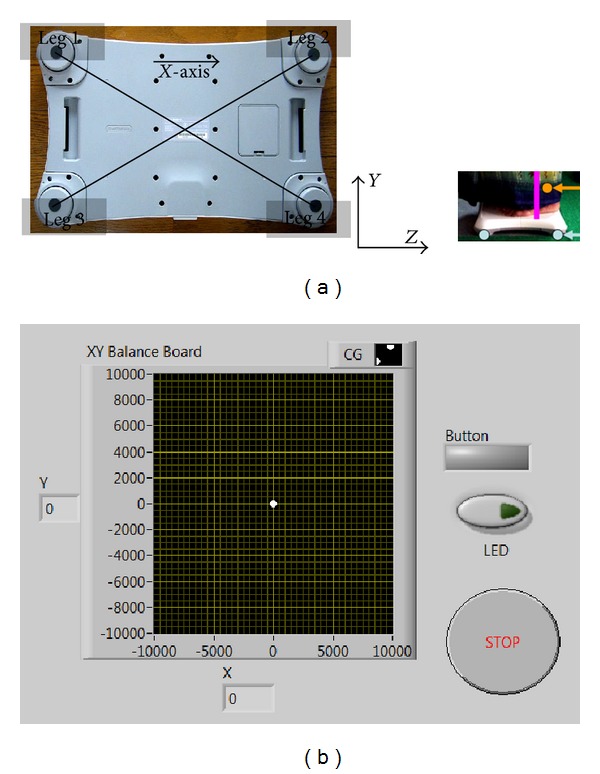
(a) The load sensors are at the four legs of Wii Balance Board (Wii BB). The origin for Wii BB reference frame was placed on the horizontal standing surface of Wii BB, which was taken at the centre of the four legs as illustrated by the intersection of the diagonals, and the *X*-direction was assumed from the centre of leg 1 to the centre of leg 2, as shown. The *Y*-direction was upwards from the horizontal standing surface of Wii BB. (b) Real-time feedback of the center of pressure location.

Wii BB was interfaced with the PC via Bluetooth using the WiiBrew toolkit [14]. The Wii BB has a useable surface of 45 cm × 26.5 cm for standing which was calibrated by placing a variety of known loads at different positions [13]. Wii BB uses four load sensors at the four legs (shown in Figure 2(a)) to measure the weight. Each sensor at each leg of the Wii BB only measures the vertical component of the force. The CoP can be approximated by interpolating the four vertical forces which was then displayed to the user, as shown at Figure 2(b). The *X*, *Y* coordinates of CoP were calculated from the streaming data using the following equation:

(1) $$\begin{array}{c} \text{CoP}\left(X,Y\right)=\frac{\sum_{i=1}^{4}\text{Weigh}{\text{t}}_{i}\left(x,y\right)}{\sum_{i=1}^{4}\text{Weigh}{\text{t}}_{i}}, \end{array}$$

where Weight_*i*_ (*x*, *y*) are the coordinates of the *i*th load sensor in the Wii BB's reference frame, Weight_*i*_ is the force recorded by the *i*th load sensor, and CoP (*X*, *Y*) are the coordinates of the CoP in the Wii BB's reference frame. Also, the BBS score was collected to identify those individuals who were classified as “fallers” (BBS below 45) and those individuals who were classified as “nonfallers” (BBS 45 or above).

#### 2.2.2. Offline Analysis of Baseline Resting State EEG

Emotiv EEG Neuroheadset wirelessly relayed EEG data to the PC from 14 channels (International 10–20 system)—AF3, AF4, F3, F4, F7, F8, FC5, FC6, P3 (CMS), P4 (DRL), P7, P8, T7, T8, O1, and O2. Resting state EEG was recorded in eyes-open and eyes-closed conditions during quiet standing for at least 3 minutes. The EEG data were analysed using EEGlab software [15]. The eye-blink artifacts were rejected using EEGlab functions, then the artifact-free continuous EEG was divided into 4.096 seconds epochs using a Hanning time window (epochs were overlapped by 50%), and an estimation of the power spectra was found for the absolute theta (4–7.5 Hz), alpha (7.5–14 Hz), and beta (14–20 Hz) frequency bands for QEEG analysis [16]. 

Following Grin-Yatsenko and colleagues [16], a three-way analysis of variance (“anovan” in MATLAB R2010a, The MathWorks, Inc., USA) of the absolute EEG power in eyes-closed condition was conducted with the following factors: GDS score (three levels: 0–9, 10–19, 20–30), EEG electrode locations (14 levels), and frequency band (three levels: theta, alpha, and beta). We then performed cluster analysis (“clusterdata” in Matlab R2010a, The MathWorks, Inc., USA) to compute the Euclidean distances between items in the data set and create a hierarchical cluster tree from the data set. Also, Linear Discriminant Classification (“ClassificationDiscriminant.fit” in Matlab R2010a, The MathWorks, Inc., USA) was investigated to evaluate the possibility of classification of depressive patients using eyes-closed EEG power spectra at discriminatory frequency bands and electrode locations, as found using multiple comparison tests (“multcompare” in Matlab R2010a, The MathWorks, Inc., USA).

#### 2.2.3. Offline Analysis of Psychomotor Symptoms

A modified functional reach task was used to quantify the subjects' ability to volitionally shift their CoP position as quickly as possible without losing balance while cued with CoP feedback via a graphical user interface (GUI) on PC, as shown in Figures 1 and 2. This modified functional reach task (FRT) resembled the Functional Reach Test, which has been shown to predict risk of falls [17]. During the FRT, the subjects were asked to keep their body rigid and to maintain full feet contact with the Wii BB, while leaning as far as possible from forward, backward, and to right and left sides, as cued by the GUI. The EEG was recorded in eyes-open condition during the consecutive FRT as well as during quiet standing (at least 3 minutes) in-between the FRT. The PT/OT conducting the FRT reported BBS and GDS scores of each elderly subject from a preliminary cross-sectional study (Section 2.1).

The EEG data from 14 channels (International 10–20 system), AF3, AF4, F3, F4, F7, F8, FC5, FC6, P3 (CMS), P4 (DRL), P7, P8, T7, T8, O1, O2, was analysed using EEGlab software [15]. The eye-blink artifacts were rejected using EEGlab functions, then the artifact-free continuous EEG was divided into 4.096 seconds epochs using a Hanning time window (epochs were overlapped by 50%), and the power spectra was estimated for the theta, alpha, and beta frequency bands for QEEG analysis [18]. The evolution of the baseline-normalized EEG power in different frequency bands (normalized by their corresponding baseline eyes-open value) during quiet standing in-between FRT was investigated for different GDS scores, EEG electrode locations, and baseline-normalized cued response time. The cued response time represented latency from visual cue to correct CoP motion out of the 95% confidence boundary of the resting-state CoP cluster in the cued direction—forward, backward, right side, and left side.

## 3. Results 

### 3.1. Preliminary Cross-Sectional Study

**Figure 3:**
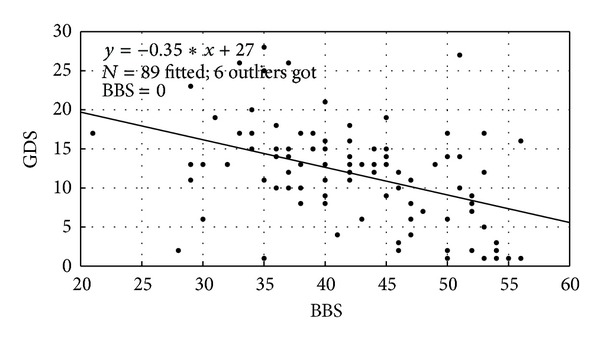
Scatter plot between Berg Balance Scale (BBS) and Geriatric Depression Scale (GDS) scores.

The descriptive statistics for age were *N* = 95, range = 60–89 years, mean = 70.12 years, std. deviation = 7.27 years. The correlation analysis between age, BBS, and GDS were performed using Pearson's correlation test where Table 2 shows the correlation coefficients. BBS showed a significant negative linear correlation with GDS (*r* = −0.423) and age (*r* = −0.362). GDS had a nonsignificant positive correlation with age (*r* = −0.162) at *P* = 0.01 level. The regression analysis of GDS as the dependent and BBS as the independent variables (predictor) for the analysis of variance showed *F*-ratio = 20.260, which was higher than the critical value. Therefore the variance attributable to the regression was significant, as shown in Figure 3. Also, the regression analysis of BBS as the dependent variable and age as the independent variable showed *F*-ratio = 14.030, which was higher than the critical value. 

Independent-sample *t*-test was used to compare the differences in GDS and BBS scores between males and females.  Table 3 shows the comparative analysis which includes mean, standard deviation, and standard error of the mean of BBS and GDS scores for the males and females. The *t*-test failed to reveal a statistically significant difference between the mean of the scores for BBS of male (mean = 43.14, std. deviation = 8.127), and female (mean = 41.45, std deviation = 7.118), *t*(93) = 1.065, *P* = 0.289, *α* = 0.05. As shown in Table 3, the *t*-test also failed to reveal a statistically significant difference between the mean of the scores for GDS of male (mean = 11.02, std. deviation = 6.498) and female (mean = 12.91, std deviation = 6.030), *t*(93) = 1.461, *P* = 0.147, *α* = 0.05.

### 3.2. Point-of-Care Testing (POCT) of Psychomotor Symptoms of Depression

**Figure 4:**
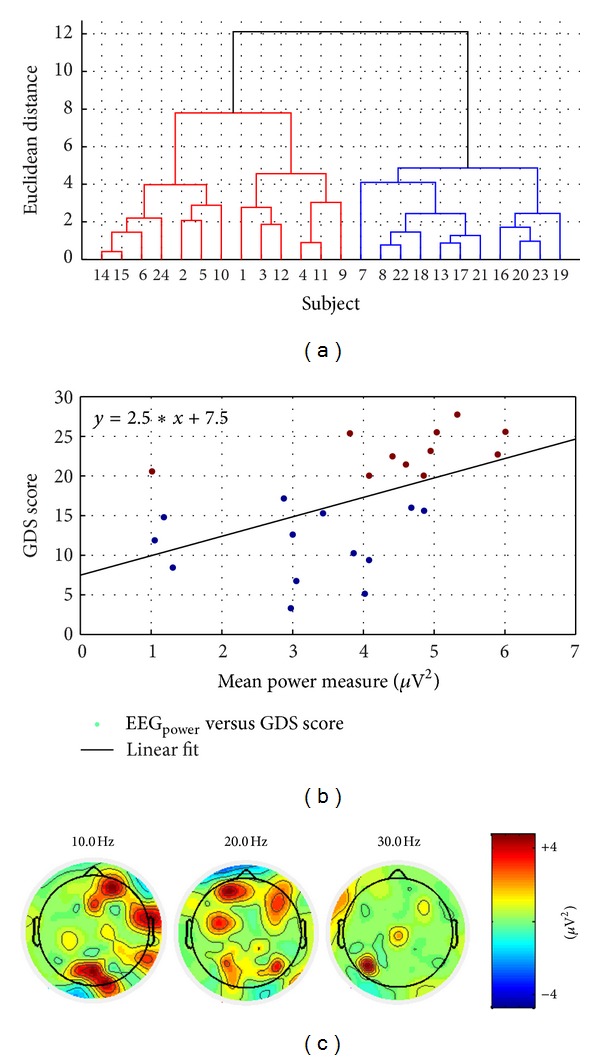
(a) Dendrogram plot of the hierarchical cluster tree from the data set—mean power measure at O1, O2, and P8 electrodes averaged over 4–30 Hz versus GDS scores. (b) Scatter plot between GDS scores and mean power measures where marker colors blue for “nondepressed” and brown for “depressed” show the two hierarchical clusters based on the Euclidean distance. (c) Topographic maps of difference EEG spectra at 10 Hz, 20 Hz, and 30 Hz between “depressed” (GDS ≥ 15) and “nondepressed” (GDS < 15) QEEG groups.

We avoided using EEG data during eyes-open condition along-with eyes-closed condition (e.g., for alpha attenuation) since substantial eye-blink artifacts appeared for eyes-open condition and not eyes-closed condition, which needed to be filtered with EEGlab. The three-way analysis of variance of absolute EEG power in eyes-closed condition with factors GDS score, EEG electrode location, and frequency bands revealed a significant (*P* < 0.01) main effect of the factors and a significant (*P* < 0.01) interaction effect between GDS score and EEG electrode location, EEG electrode location and frequency band, but nonsignificant (*P* = 0.09) interaction effect between the frequency band and the GDS score. Posthoc analysis revealed an increased spectrum power during eyes-closed condition across all frequency bands of the resting state EEG for occipital (O1, O2) and parietal (P8) electrode locations that correlated with worse depression scores.  Figure 4(a) shows the dendrogram plot of the hierarchical cluster tree from the data set—mean power measure at O1, O2, and P8 electrodes averaged over 4–30 Hz versus GDS scores. The hierarchical cluster tree reveals primarily two clusters, GDS = 0–15 and GDS = 15–30, that are shown in Figure 4(b) with marker colors blue and brown, respectively, for the scatter plot between GDS scores and mean power measures. In accordance, Linear Discriminant Classification was conducted using mean power measures at O1, O2, and P8 electrodes averaged over 4–30 Hz, where the average percent of classification was found to be >84% for “nondepressed” (GDS = 0–15) QEEG group and >92% for “depressed” (GDS = 15–30) QEEG group. 

**Figure 5:**
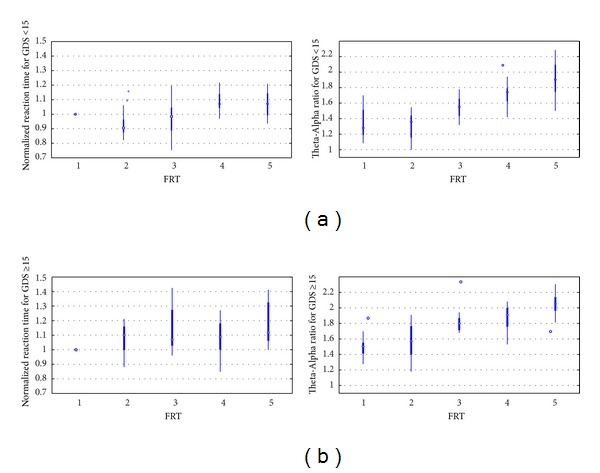
Box plot showing the evolution of baseline-normalized cued response time during modified functional reach tasks (FRT) and the Theta-Alpha power ratio during quiet standing in-between FRT for (a) “nondepressed” (GDS < 15) QEEG group and (b) “depressed” (GDS ≥ 15) QEEG group.

**Figure 6:**
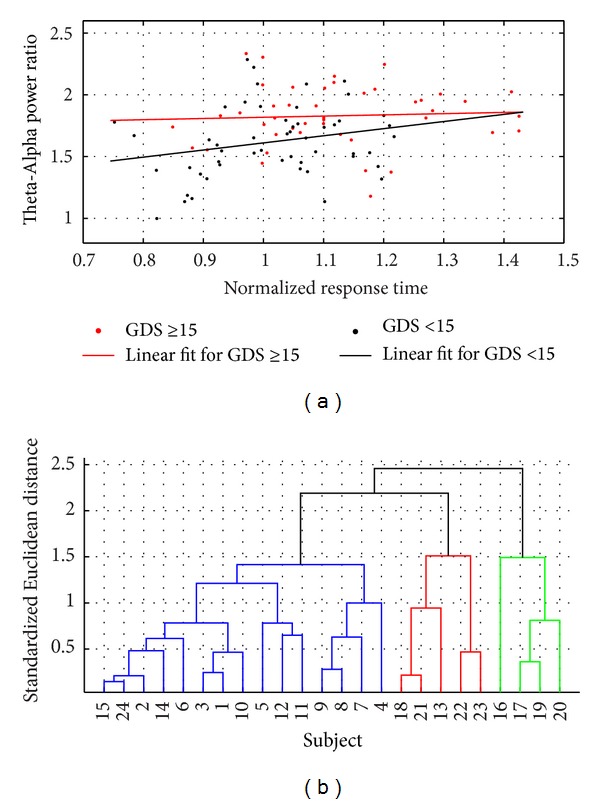
(a) Scatter plot between baseline-normalized cued response time and the Theta-Alpha power ratio, and linear regression fit for the “nondepressed” (GDS < 15) and “depressed” (GDS ≥ 15) QEEG groups. (b) Dendrogram plot of the hierarchical cluster tree from the data set—average slope of the Theta-Alpha power ratio versus average slope of baseline-normalized cued response time—providing three clusters shown with blue, red, and green colored linkages.

During the modified functional reach task (FRT), a quicker transition to mental fatigue [18] was found in the “depressed” (GDS ≥ 15) QEEG group as elucidated by a faster increase in the theta band (4–8 Hz) baseline-normalized power in the frontal (F3, F4) electrodes and a faster decrease in the alpha band (8–13 Hz) baseline-normalized power in the parietal (P8) electrodes. Therefore, we defined Theta-Alpha power ratio as a QEEG marker of fatigue, which is the ratio of mean theta band baseline-normalized power in frontal (F3, F4) electrodes to mean alpha band baseline-normalized power in the parietal (P8) electrodes.  Figure 5(a) shows the evolution of baseline-normalized response time during FRT and the Theta-Alpha power ratio during quiet standing in-between the FRT in “nondepressed” (GDS < 15) QEEG group while Figure 5(b) shows the same in “depressed” (GDS ≥ 15) QEEG group. In fact, there was a slight initial decrease in the response time in the “nondepressed” (GDS = 0–15) QEEG group before long-term increase due to fatigue, which may be due to initial selective attention to task performance. It was found that the increase in baseline-normalized response time during FRT was quicker in the “depressed” (GDS ≥ 15) QEEG group. Indeed, the baseline-normalized response time correlated with the Theta-Alpha power ratio with Pearson's linear correlation coefficient, *r* = 0.2631 at *P* = 0.0096 level. Also, Pearson's linear correlation coefficient for the “nondepressed” (GDS < 15) QEEG group was *r* = 0.2318 at *P* = 0.0631 level and for the “depressed” (GDS ≥ 15) QEEG group was *r* = 0.2160 at *P* = 0.1131 level, as shown in Figure 6(a). Accordingly, the dendrogram plot of the hierarchical cluster tree from the data set—average slope of the Theta-Alpha power ratio versus average slope of baseline-normalized response time—provided three clusters as shown in Figure 6(b) with blue, red, and green colors, which compared well with the dendrogram plot of the hierarchical cluster tree in Figure 4(a) (compare subject number). 

## 4. Discussion 

In this preliminary cross-sectional study, balance (BBS) score had a significant negative linear correlation with depression (GDS) score, which implied that as the depression increased, balance decreased, and vice versa. Since balance (BBS) score also showed a significant negative linear correlation with age, so depression (GDS) score could have been affected by age via balance (BBS) score; however, depression (GDS) score was found to only have a nonsignificant positive correlation with age. Although correlation does not mean causality, but these findings are consistent with those of Kose and colleagues [19], who found that depressive and cognitive symptoms, insufficient mobility, balance impairment and other risk factors are related to each other. In fact, there is a vicious cycle among these parameters. Rochester and colleagues [20] found that cognitive function, depression, physical fatigue, and balance were significantly related to walking speed for functional tasks. These findings are further consistent with those of Suárez and colleagues [21] who found that age-related changes in the neural, sensory, and musculoskeletal systems can lead to balance impairments that affect the ability to move around safely. Newton [22] found through multiple regression analysis that the frequency of performing activities and the comfort in performing activities without the fear of falling significantly contributed to the scores on the balance tests. The probable reason for such a finding may be that balance and depression are both the main risk factors for falls. These findings are consistent with those of Sai and colleagues [23] who had reported many risk factors for falls including a history of falls, lower extremity weakness, balance and gait abnormalities, decreased muscle strength, old age, cognitive impairment, medications, orthostatic hypotension, anemia, female gender, arthritis, and psychological factors including unipolar and bipolar depression and manic depressive disorder. Here, one limitation of our cross-sectional study design was that we did not collect retrospective data on the history of falls. We have now developed an Android application, FallAlert (Gnan Systems LLP, India) [24] to monitor falls using a mobile phone in our future studies.

The QEEG analysis of resting-state EEG showed increased spectrum power across all frequency bands for occipital (O1, O2) and parietal (P8) electrodes that correlated with worse depression (GDS) scores. However, eyes-closed EEG power spectra could reveal only two groups, GDS = 0–15 and GDS = 15–30, where mildly depressed (GDS = 10–19) patients were included either in “nondepressed” (GDS = 0–15) or “depressed” (GDS = 15–30) QEEG groups, making them a mix group. It was possible to classify subjects with only <16% false positive rate and >92% true positive rate using eyes-closed EEG power spectra. Here, monitoring QEEG along with the cued response time during modified functional reach tasks (FRTs) may provide insights into the psychomotor symptoms of depression where a quicker increase in the cued response time and Theta-Alpha power ratio was found in the “depressed” (GDS > 15) QEEG group, which implied a quicker transition to fatigue. In “nondepressed” (GDS ≤ 15) QEEG group there was a slight decrease in the Theta-Alpha power ratio from the baseline resting-state before a long-term increase, which may be attributed to acute effects due to selective attention related to task performance [25]. However in long-term, there was a faster increase in theta band baseline-normalized power in frontal electrodes and a faster decrease in alpha band baseline-normalized power in the parietal electrodes in the “depressed” (GDS > 15) QEEG group. In fact, decreased alpha power activity has been shown to be related to sleepiness, and a decreased high-level information processing is associated with decreased alpha power densities [26]. Therefore, a biomarker of the psychomotor symptoms of depression was investigated using the hierarchical cluster tree from the data set—average slope of the Theta-Alpha power ratio versus average slope of baseline-normalized response time, which provided three clusters that remain to be evaluated in future clinical studies using the motor agitation and retardation scale (MARS) [27]. Nevertheless, we found a good correspondence with the QEEG group where the biomarker of fatigue during FRT was better at discriminating the mild depression and severe depression (compare subject number in Figures 4(a) and 6(b)). Once validated with MARS, the biomarker can be used to plan pharmacological interventions targeting psychomotor symptoms of depression.

The distinctive feature of our study was in monitoring QEEG along with the cued response time during FRT, which provided insights into the psychomotor symptoms of depression. Here, the POCT system may also be used to provide real-time feedback and monitoring of the biomarker, where a comprehensive therapy program can be developed using functional reach training in conjunction with pharmacological interventions. Furthermore, the frequency of falls can be monitored with a freely available mobile phone-based application, FallAlert [24], during such a comprehensive therapy program where the propensity of falls during periods of psychomotor symptoms of depression in elderly can be investigated in an interventional study.

## Figures and Tables

**Table 1:** Specifications of the Emotiv EEG Neuroheadset [12].

Number of channels	14 (plus CMS/DRL references)
Channel names (Int. 10–20 locations)	AF3, AF4, F3, F4, F7, F8, FC5, FC6, P3 (CMS), P4 (DRL), P7, P8, T7, T8, O1, O2
Sampling method	Sequential sampling, single ADC
Sampling rate	~128 Hz (2048 Hz internal)
Resolution	16 bits (14 bits effective) 1 LSB = 0.51 *μ*V
Bandwidth	0.2–45 Hz, digital notch filters at 50 Hz and 60 Hz
Dynamic range (input referred)	256 mVpp
Coupling mode	AC coupled
Connectivity	Proprietary wireless, 2.4 GHz band
Battery type	Li-poly
Battery life (typical)	12 hrs
Impedance measurement	Contact quality using patented system

**Table 2:** Correlation between age, BBS, and GDS.

		Age	BBS	GDS
Age	Pearson's correlation	1	−0.362**	0.162
	Sig. (2-tailed)		0.000	0.116

BBS	Pearson's correlation	−0.362**	1	−0.423**
	Sig. (2-tailed)	0.000		0.000

GDS	Pearson's correlation	0.162	−0.423**	1
	Sig. (2-tailed)	0.116	0.000	

**Correlation is significant at the *P* = 0.01 level (2-tailed).

**Table 3:** Comparison between male and female with BBS and GDS.

	Gender	*N* =	Mean	Std. deviation	Std. error mean
BBS	Male	51	43.14	8.127	1.138
	Female	44	41.45	7.118	1.073

GDS	Male	51	11.02	6.498	0.910
	Female	44	12.91	6.030	0.909

## References

[1] Pollock BG, Reynolds CF (2000). Depression late in life. *The Harvard Mental Health Letter*.

[2] Lebowitz BD, Pearson JL, Schneider LS (1997). Diagnosis and treatment of depression in late life: consensus statement update. *Journal of the American Medical Association*.

[3] http://planningcommission.nic.in/aboutus/committee/wrkgrp12/health/WG_3_2non_communicable.pdf.

[4] Zijlstra GAR, van Haastregt JCM, van Eijk JTM, van Rossum E, Stalenhoef PA, Kempen GIJM (2007). Prevalence and correlates of fear of falling, and associated avoidance of activity in the general population of community-living older people. *Age and Ageing*.

[5] Sobin C, Sackeim HA (1997). Psychomotor symptoms of depression. *American Journal of Psychiatry*.

[6] Kumar R, Aikat R, Banerjee A Relationship between balance and depression in elderly.

[7] Berg K (1989). Measuring balance in the elderly: preliminary development of an instrument. *Physiotherapy Canada*.

[8] Yesavage JA, Brink TL, Rose TL (1982). Development and validation of a geriatric depression screening scale: a preliminary report. *Journal of Psychiatric Research*.

[9] Dutta A, Banerjee A, Dutta A Low-cost visual postural feedback with Wii balance board and Microsoft Kinect—a feasibility study.

[10] Ganguli M, Dube S (1999). Depressive symptoms, cognitive impairment and functional impairment in a rural elderly population in India. *International Journal of Geriatric Psychiatry*.

[11] Ganguli M, Ratcliff G, Chandra V (1995). A Hindi version of the MMSE: the development of a cognitive screening instrument for a largely illiterate rural elderly population in India. *International Journal of Geriatric Psychiatry*.

[12] http://emotiv.wikia.com/wiki/Emotiv_EPOC.

[13] Koslucher F, Wade MG, Nelson B, Lim K, Chen FC, Stoffregen TA (2012). Nintendo Wii Balance Board is sensitive to effects of visual tasks on standing sway in healthy elderly adults. *Gait Posture*.

[14] http://wiibrew.org/wiki/Wii_Balance_Board.

[15] Delorme A, Makeig S (2004). EEGLAB: an open source toolbox for analysis of single-trial EEG dynamics including independent component analysis. *Journal of Neuroscience Methods*.

[16] Grin-Yatsenko VA, Baas I, Ponomarev VA, Kropotov JD (2009). EEG power spectra at early stages of depressive disorders. *Journal of Clinical Neurophysiology*.

[17] Duncan PW, Studenski S, Chandler J, Prescott B (1992). Functional reach: predictive validity in a sample of elderly male veterans. *Journals of Gerontology*.

[18] Hankins TC, Wilson GF (1998). A comparison of heart rate, eye activity, EEC and subjective measures of pilot mental workload during flight. *Aviation Space and Environmental Medicine*.

[19] Kose N, Cuvalci S, Ekici G, Otman AS, Karakaya MG (2005). The risk factors of fall and their correlation with balance, depression, cognitive impairment and mobility skills in elderly nursing home residents. *Saudi Medical Journal*.

[20] Rochester L, Hetherington V, Jones D (2004). Attending to the task: interference effects of functional tasks on walking in Parkinson’s disease and the roles of cognition, depression, fatigue, and balance. *Archives of Physical Medicine and Rehabilitation*.

[21] Suárez H, Suárez A, Lavinsky L (2006). Postural adaptation in elderly patients with instability and risk of falling after balance training using a virtual-reality system. *International Tinnitus Journal*.

[22] Newton RA (1997). Balance screening of an inner city older adult population. *Archives of Physical Medicine and Rehabilitation*.

[23] Sai AJ, Gallagher JC, Smith LM, Logsdon S (2010). Fall predictors in the community dwelling elderly: a cross sectional and prospective cohort study. *Journal of Musculoskeletal Neuronal Interactions*.

[24] https://play.google.com/store/apps/details?id=com.gnansys.fallalert.

[25] Boksem MAS, Meijman TF, Lorist MM (2005). Effects of mental fatigue on attention: an ERP study. *Cognitive Brain Research*.

[26] Stampi C, Stone P, Michimori A (1995). A new quantitative method for assessing sleepiness: the alpha attenuation test. *Work and Stress*.

[27] Sobin C, Mayer L, Endicott J (1998). The motor agitation and retardation scale: a scale for the assessment of motor abnormalities in depressed patients. *Journal of Neuropsychiatry and Clinical Neurosciences*.

